# MicroRNA expression in JAG1/Notch-activated periodontal ligament stem cells

**DOI:** 10.1038/s41405-024-00232-5

**Published:** 2024-06-05

**Authors:** Promphakkon Kulthanaamondhita, Chatvadee Kornsuthisopon, Ajjima Chansaenroj, Vorapat Trachoo, Jeeranan Manokawinchoke, Lakshman Samaranayake, Supreda Suphanantachat Srithanyarat, Thanaphum Osathanon

**Affiliations:** 1 Center of Excellence for Dental Stem Cell Biology, Faculty of Dentistry, Chulalongkorn University, Bangkok, Thailand; 2 Department of Anatomy, Faculty of Dentistry, Chulalongkorn University, Bangkok, Thailand; 3 Department of Oral and Maxillofacial Surgery, Faculty of Dentistry, Chulalongkorn University, Bangkok, Thailand; 4 Faculty of Dentistry, University of Hong Kong, Pok Fu Lam, 34, Hospital Road, Hong Kong; 5 Dean Office and Office of Research Affairs, Faculty of Dentistry, Chulalongkorn University, Bangkok, Thailand; 6 Department of Periodontology, Faculty of Dentistry, Chulalongkorn University, Bangkok, Thailand; 7 Center of Excellence for Periodontology and Dental Implants, Department of Periodontology, Faculty of Dentistry, Chulalongkorn University, Bangkok, Thailand

**Keywords:** Periodontics, Resorption

## Abstract

**Objectives:**

The study explored the expression profile of miRNAs in Notch-activated periodontal ligament stem cells (PDLSCs) and examined their potential cellular targets.

**Methods:**

PDLSCs were cultured and treated with indirect immobilized Jagged1. The miRNA expression profile was examined using NanoString analysis. Bioinformatic analysis was performed together with enrichment, and miRNA expression was evaluated and validated using a quantitative polymerase chain reaction (qPCR).

**Results:**

A total of 26 miRNAs were differentially expressed in Jagged1 treated PDLSCs compared with the controls. Pathway analysis revealed that altered miRNAs were significantly associated with the transforming growth factor β (TGF-β) signaling pathway. Target prediction analysis demonstrated that 11,170 genes as predictable targets of these altered miRNAs. Enrichment of predicted target genes revealed that they were related to ErbB, Ras and MAPK signaling pathways and small GTPase transduction.

**Conclusions:**

The research concludes that several miRNAs are differentially expressed in jagged-1 treated PDLSCs. In translational terms the differential functionality of these miRNAs offer promise for the development of targeted regenerative materials that are necessary for managing lost tissue replacement in periodontal diseases.

## Introduction

Notch signaling pathway was first discovered in the fruit fly Drosophila [1]. This signaling pathway plays an important role in the cell-cell communication and participates in the regulation of numerous biological processes from embryonic to adult life including organ developments, pathogenesis, and regeneration [2, 3]. Previous studies have investigated the role of Notch signaling in the promotion of odonto/osteogenic differentiation in various cell types including human periodontal ligament stem cells [4, 5]. Moreover, in vitro studies have shown that the overexpression of Notch ligand, Jagged1, in human mesenchymal stem cells (MSCs) upregulated the enzymatic activity of alkaline phosphatase and enhanced mineral deposition [6, 7].

Periodontal ligament (PDL) is a specialized tissue which connects the root cementum with the alveolar bone and helps the tooth to resist and mitigate the chewing forces [8]. Previous studies have demonstrated that human periodontal ligament cells (PDLCs) constitute a subpopulation of stem cells exhibiting distinctive features, such as self-renewal and multi-lineage differentiation potential. These cells are commonly referred to as periodontal ligament stem cells (PDLSCs) [9–11]. The association between Notch signaling and PDLSCs during osteogenic differentiation has been investigated. Upon Jagged-1 treatment, the mRNA and protein levels of osteoprotegerin (OPG) significantly decreased, whereas receptor activator of nuclear factor kappa-B ligand (RANKL) remained unchanged [12]. Furthermore, bioinformatic analysis has also revealed that Jagged1 promotes the expression of various genes associated with osteoblast differentiation-related gene ontology, including alkaline phosphatase (*ALPL*), which generates free phosphate and subsequently co-precipitates with calcium ions, thereby promoting mineralization [5].

MicroRNAs (miRNAs) are one of the molecules involved in the regulation of biological processes of the cells through the degradation of mRNA. MiRNAs have been shown to modulate the stemness and differentiation of various types of mesenchymal stem cells including PDLSCs [13, 14]. During the differentiation of human PDLSCs stimulated by various osteogenic conditions, the differential expression of miRNAs was observed. These miRNAs have been found to play an essential role by targeting osteogenic markers and related pathways [15, 16].

While the treatment with Jagged1 significantly enhances osteogenesis in human PDLSCs, the underlying biological mechanisms are yet to be investigated. With the current understanding of miRNA function, it could be speculated that Jagged1 might partly modulate the osteogenesis of PDLSCs via the regulation of several miRNAs. Therefore, the current investigation aimed at demystifying the miRNA expression profile and the potential regulatory mechanisms in Jagged1-treated PDLSCs.

## Methods

### Cell isolation and culture

Cell isolation protocols were approved by the Human Research Ethics Committee, Faculty of Dentistry, Chulalongkorn University (approval no.123/2022). The experimental protocol was approved by the Institutional Biosafety Committee of the Faculty of Dentistry, Chulalongkorn University, (Dent-CU-IBC) (approval no.006/2023). Prior to tooth extraction, all patients were informed regarding the research protocol and provided signed consent forms.

A total of four human, third molars surgically extracted due to impactions, at the Oral Surgery Clinic, Faculty of Dentistry, Chulalongkorn University were used to obtain PDL tissues. The teeth were thoroughly washed with phosphate buffered saline (PBS), and the PDL tissues were scraped from the middle third of the root surface with a blade and scalpel under sterile conditions. These explants were then grown in Dulbecco’s modified eagle medium (DMEM) (Gibco, USA) supplemented with 10% fetal bovine serum (Gibco, USA), 2 mM L-glutamine, 100 units/ml penicillin, 100 g/ml streptomycin, and 250 ng/ml amphotericin B (Gibco, USA) in a humidified atmosphere with 5% CO_2_ at 37 °C for at least 20 days before subculture as per the method of Manokawinchoke et al. [17]. The culture medium was changed every 3-4 days. After reaching confluence, cells were subcultured with 0.25% Trypsin-EDTA (Gibco, USA). Cells from 3rd to 5th passage were used throughout this study.

### Flow cytometry

Flow cytometry was employed to assess the surface protein expression in PDLSCs. Following cell harvesting, a single-cell suspension was prepared. Subsequently, the harvested cells underwent staining using fluorescence-conjugated antibodies: PE-conjugated anti-human CD105 (Immuno Tools, Germany), FITC-conjugated anti-human CD44 (BD Bioscience, USA), PerCP-conjugated anti-CD45 (Abcam, USA), and FITC-conjugated anti-CD90 (Abcam, USA) at a 1:50 dilution. Mean fluorescence intensity was determined using a FACSCalibur flow cytometer and CellQuest software (BD Bioscience, USA).

### Differentiation assay

For osteo/odontogenic differentiation, cells were seeded into 24-well plates (25,000 cells/well) and maintained in the aforementioned growth medium for 24 h, and subsequently, the medium was changed to a differentiation induction medium. The latter osteogenic induction medium constituents included 50 µg/mL ascorbic acid (Sigma-Aldrich, USA), 250 nM dexamethasone (Sigma-Aldrich, USA), and 5 mM β-glycerophosphate (Sigma-Aldrich, USA). After 14 days of culture, the induced mineral deposition was evaluated using alizarin red staining.

For the adipogenic differentiation assays, an induction medium was prepared with 1 μM dexamethasone (Sigma-Aldrich, USA), 0.2 mM indomethacin (Sigma-Aldrich, USA), 1 mM IBMX (Thermo Fisher Scientific, USA), and 0.1 mg/ml insulin (Sigma-Aldrich, USA). Following a 16-day induction period, intracellular lipid droplet accumulation was evaluated using oil-red O staining.

### Jagged1 treatment

The immobilized indirect Jagged1 treatment was performed according to our previous study [12]. Briefly, recombinant protein G (Sigma-Aldrich, USA) was incubated in a tissue culture plate at a concentration of 50 μg/mL for 16 h. The surface was then incubated with 10 mg/mL of BSA (Sigma-Aldrich, USA) for 2 h followed by incubation with Jagged1/Fc (R&D systems, USA) for 2 h. An equal volume of human IgG, the Fc fragment (Sigma-Aldrich, USA), was used as a control. The tissue culture surface was washed three times with sterile PBS between each step. Prior to cell seeding in further experiments, the culture surfaces were washed once with culture media.

### NanoString analysis

Total RNAs were isolated using an miRNeasy Kits (Qiagen, Germany). RNA concentration and purification were assessed by using NanoDrop (Thermo Scientific, USA). For each sample, 100 ng of total RNA were subjected to NanoString analysis of miRNA expression at the Omics and Bioinformatics Center, Faculty of Science, Chulalongkorn University. The miRNA expression was then analyzed using nSolver software (NanoString Technologies, USA) to determine the differentially expressed miRNA.

### Bioinformatic analysis

Bioinformatic analysis was performed using the Reactome database with an adjusted false discovery rate of 0.05. Afterwards the target signaling interactions were examined. A heatmap of significantly altered miRNAs due to target signaling was generated using Heatmapper software (Wishart Research Group, Canada) [18]. The predicted pathways and gene interaction were explored using miRPath V.3 [19], TargetScanHuman 8.0 [20] and miRWalk [21] databases. The associated biological functions of the predicted genes were assessed using WebGestalt (WEB-based Gene SeT AnaLysis Toolkit) [22].

### Real-time polymerase chain reaction (RT-PCR)

TRIzol reagent (Invitrogen, USA) was used to facilitate a total cellular RNA extraction. The NanoDrop (Thermo Scientific, USA) was utilized to determine RNA concentration and integrity. Subsequently, 1 microgram of total RNA was converted to complementary DNA (cDNA) using the miRCURY LNA RT Kit (Qiagen, Germany), followed by real-time PCR using the miRCURY LNA SYBR Green PCR Kit (Qiagen, Germany). miRNA primers were purchased from Qiagen (Germany), and reactions were conducted on the MiniOpticon real-time PCR system (Bio-Rad, USA). U6 snRNA was used for miRNA level normalization, and relative miRNA expression quantification was performed using the comparative Ct method (2^−ΔΔCt^ method) [23].

### Statistical analyses

All data are represented as mean ± SD. Each dot on the graph indicates the raw values. The Mann Whitney U test was used for a two-group comparison. A *p*-value of less than 0.05 was considered statistically significant. Data were analyzed using GraphPad Prism 8.0 (USA). All experiments were performed in triplicate.

## Results

### Stem cell characteristics of Jagged1-treated PDLSCs

**Fig. 1:**
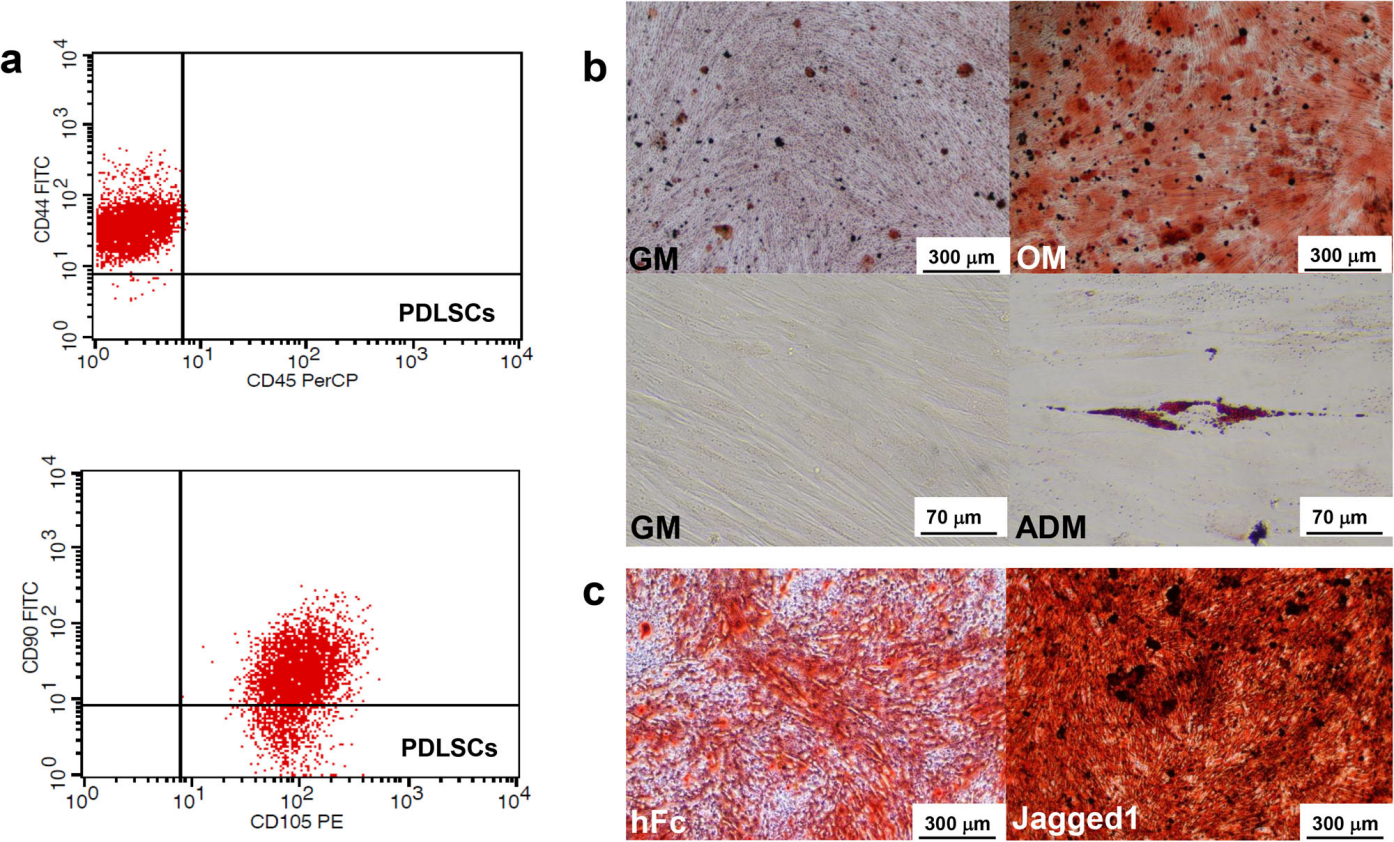
Characterization of the human PDL-derived mesenchymal stem cells. Representative histogram indicating expression of MSCs markers as evaluated by flow cytometry (**a**). Multilineage differentiation of the osteo/odontogenic and adipogenic lineages examined by alizarin red s staining and oil red o staining (**b**). Cells were seeded on Jagged1 immobilized surface and maintained in osteo/odontogenic medium. Cells on hFc-immobilized surfaces were used as controls. Osteo/odontogenic differentiation was evaluated in vitro using alizarin red s staining (**c**).

Cells isolated from human periodontal tissues exhibited stem cell features as demonstrated by positive staining of CD44, CD90 and CD105 (Fig. 1a). The osteogenic and adipogenic differentiation potential of these cells were also demonstrated via mineral deposition and intracellular lipid accumulation, respectively (Fig. 1b). Jagged1-treated PDLSCs exhibited greater potential for mineral deposition compared to the control hFc group, after maintenance in the osteogenic induction medium for 14 days (Fig. 1c).

### miRNAs expression profile and predicted target pathways

**Fig. 2:**
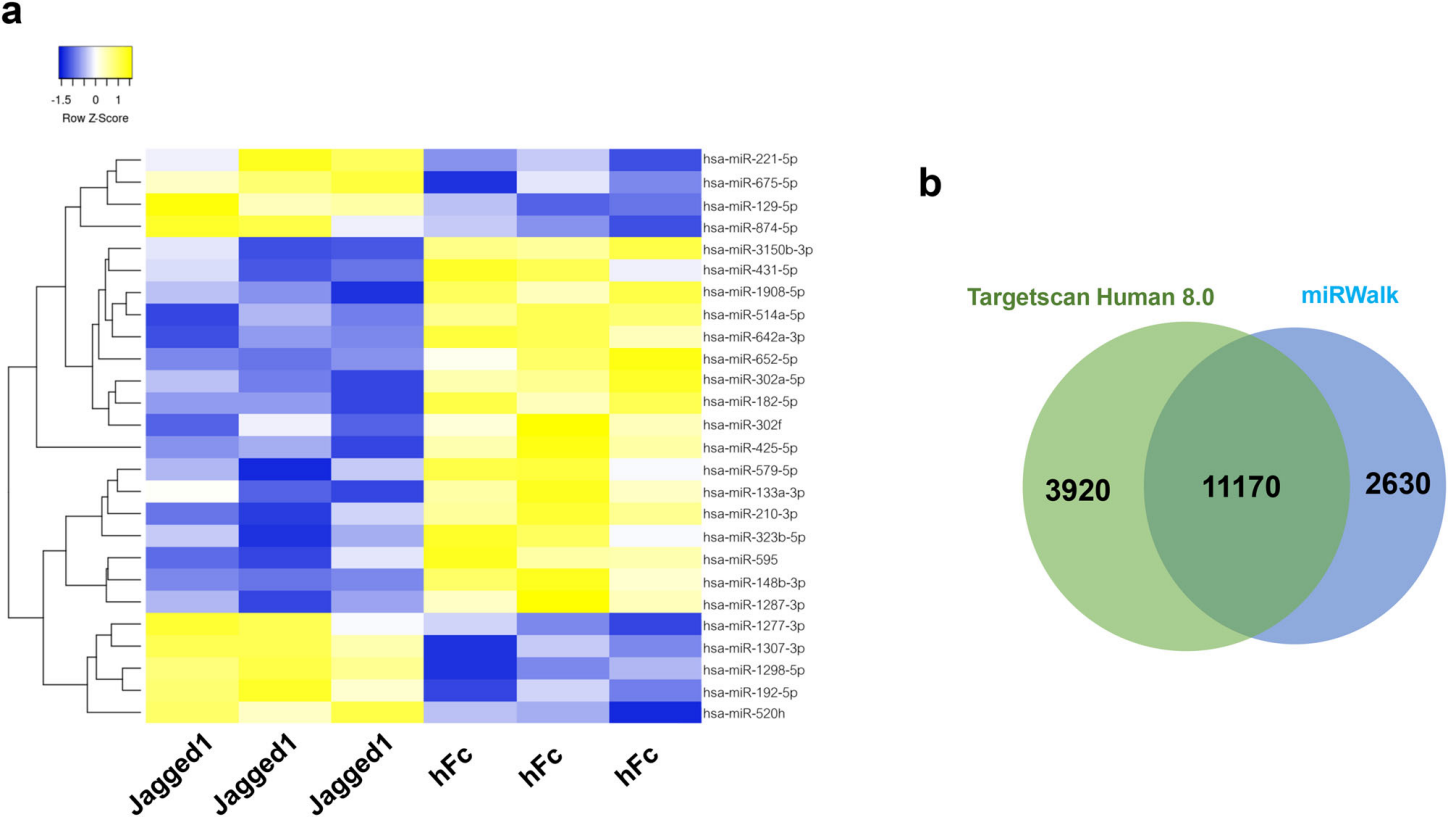
Significantly altered miRNAs and their predicted target genes in Jagged-1 treated PDLSCs. Heatmap of significantly alter miRNAs in Jagged1-treated PDLSCs (**a**). Venn diagram of predicted target genes of the significantly altered miRNAs (**b**).

The expression profiling data from the NanoString analysis demonstrated 26 differentially expressed miRNA in Jagged1-treated PDLSCs (Fig. 2a). Among these altered miRNAs, 9 miRNAs were upregulated whereas the remaining 17 miRNAs were downregulated. MiRPath software predicted the Top 10 signaling pathways regulated by these significantly altered miRNAs (Table 1) and revealed that these miRNAs were involved with several biological pathways including extracellular matrix (ECM)-receptor interaction and transforming growth factor-β (TGF-β) signaling pathway implying that the osteogenesis process of PDLSCs is likely to be modulated by these altered miRNAs.

**Table 1:** Top 10 signaling pathways associated with significantly associated with altered miRNAs.

Pathway	*p*-value
ECM-receptor interaction	1.03 × 10^−8^
Prion diseases	6.11 × 10^−7^
TGF-β signaling pathway	5.54 × 10^−6^
Lysine degradation	2.37 × 10^−5^
Proteoglycans in cancer	2.42 × 10^−5^
Pathways in cancer	5.91 × 10^−5^
Nicotine addiction	7.30 × 10^−5^
Phosphatidylinositol signaling system	< 0.01
Renal cell carcinoma	< 0.01
Axon guidance	< 0.01

### MiRNAs and their involvement in the cellular biological processes and regulation of molecular functions

**Fig. 3:**
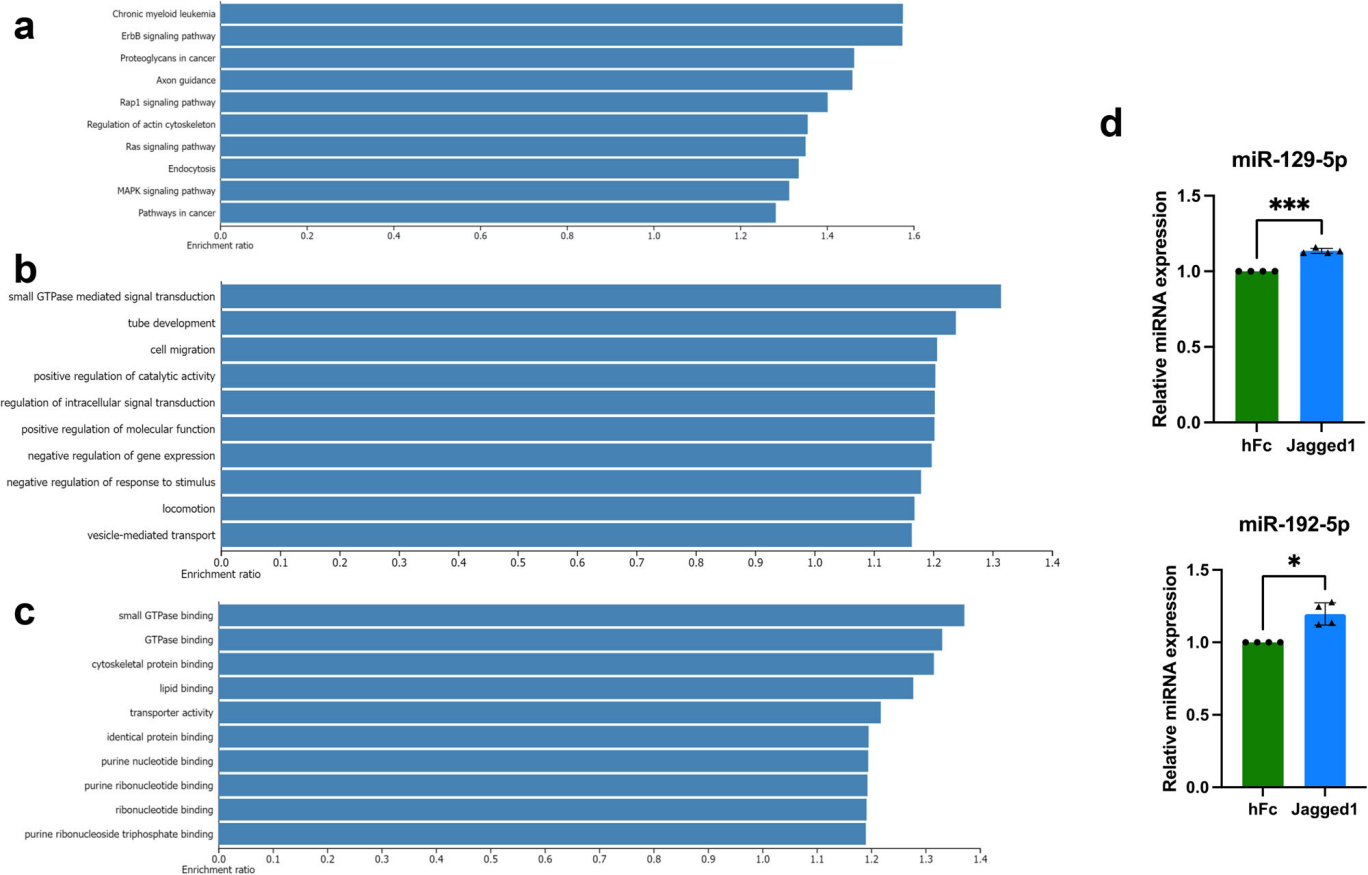
Bioinformatics analysis of the predicted target genes and validation of the target miRNA. GO analysis of the predicted target genes (**a**). Molecular functions associated with the predicted target genes of the altered miRNAs (**b**) and biological processes associated with the predicted target genes of the altered miRNAs (**c**). RT-PCR analysis of the representative miRNAs (**d**). The results are shown in mean ± SD (*n* = 4).

TargetScanHuman 8.0 and miRWalk databases were used to predict potential target genes of significantly altered miRNAs. There were 11,170 common genes that were predicted to be the targets of these altered miRNAs (Fig. 2b). Enrichment analysis of the predicted genes from the significantly altered miRNAs was performed using the Web Gestalt. Gene ontology (GO) analysis of the predicted target genes showed the enrichment of several biological pathways, including ErbB, Rap1, Ras and MAPK signaling pathways (Fig. 3a).

The regulation of molecular functions and cellular biological processes of the predicted genes was enriched. These predicted genes were involved in the binding of purine nucleotides and ribonucleotides and were correlated with the binding of small GTPases. Moreover, these genes also play a part in small GTPase mediated signal transduction, regulation of intracellular signal transduction and cell migration (Fig. 3b, c).

### RT-PCR analysis of the representative miRNAs

On analysis of miRNA expression profiling, various miRNAs associated with the stemness, and osteogenic differentiation of the mesenchymal stem cells were identified as being differentially expressed in Jagged1-treated PDLSCs. Accordingly, miR-129-5p and miR-192-5p were significantly altered and their expressions were validated through RT-PCR. The upregulation of these miRNAs in the Jagged1-treated PDLSCs was significantly greater in comparison to the untreated controls (*p* < 0.05: Fig. 3d). The target pathway prediction analysis demonstrated that both of the latter miRNAs enriched the signaling pathways regulating pluripotency of stem cells (Tables 2 and 3). Additionally, miR-129-5p was found to be involved in the Hippo signaling pathway, which is related to the regulation of bone homeostasis (Table 2), while miR-192-5p was associated with the Wnt signaling pathway, an important regulatory pathway in osteogenic differentiation (Table 3).

**Table 2:** Top 10 signaling pathways associated with miR-129-5p.

Pathway	*p*-value
Prostate cancer	<0.01
Hippo signaling pathway	<0.01
Viral carcinogenesis	0.01
mRNA surveillance pathway	0.03
Pathways in cancer	0.03
Renal cell carcinoma	0.03
Glioma	0.03
p53 signaling pathway	0.03
Signaling pathways regulating pluripotency of stem cells	0.04
Melanoma	0.04

**Table 3:** Top 10 signaling pathways associated with miR-192-5p.

Pathway	*p*-value
Prion diseases	3.44 × 10^−7^
Folate biosynthesis	<0.01
Wnt signaling pathway	<0.01
Steroid biosynthesis	<0.01
Lysine degradation	<0.01
Signaling pathways regulating pluripotency of stem cells	<0.01
Base excision repair	<0.01
Fanconi anemia pathway	<0.01
Cell cycle	0.01
HTLV-I infection	0.01

## Discussion

Our study demonstrated the differential expression profile of miRNAs in Jagged1-exposed PDLSCs compared with untreated control cell populations. The altered miRNAs partake in several biological pathways that regulate cellular proliferation and differentiation, notably the TGF-β signaling pathways as well as regulation of molecular functions including small GTPase binding and ErbB signaling pathways.

The small GTPase family is a GTP-binding protein commonly found in normal cells. It is intimately associated with the dynamic transition between the guanosine triphosphate (GTP)-associated active state and the guanosine diphosphate (GDP)-bound inactive state. Consequently, these proteins interact with the downstream effector proteins and regulate diverse cellular processes, including cell differentiation, growth, and proliferation [24]. In a recent study utilizing targeted proteomic profiling, Yang et al. [25] demonstrated altered expression of small GTPases during osteogenic differentiation, particularly those associated with autophagy, that eventually led to promotion of osteogenic differentiation. From these findings, it could be surmised that miRNA promotes small GTPase binding in PDLSCs, thus modulating osteogenic differentiation.

ErbB on the other hand belongs to the epidermal growth factor receptor (EGFR) family. The ErbB signaling regulates cell proliferation, apoptosis, and cell motility *via* three different pathways, the PI3K/AKT, the JAK/STAT, and the MAPK signaling pathway [26]. A previous study has shown that ErbB signaling is crucial for the conservation and maturation of chondrocyte, osteoblast proliferation, and the differentiation of periosteal osteoblasts [27]. In our study, the predicted target genes of the altered miRNAs were associated with the ErbB signaling, suggesting that miRNA may contribute to osteogenesis by modulating the ErBb signaling cascades.

Previous studies have shown that several miRNAs are involved in promoting odonto/osteoblastic differentiation in various stem cell lineages [28, 29]. In bone marrow-derived MSCs (BMMSCs), overexpression of miR-129-5p significantly enhanced osteogenic differentiation by upregulating alkaline phosphatase (ALP) activity, mineral deposition, and expression of osteogenic marker genes via Dkk3-mediated activation of the Wnt/*β*-catenin pathway [30]. Furthermore, BMMSCs cultures supplemented with serum containing high concentrations of miR-129-5p also demonstrated increased expression of *RUNX2*, collagen type I alpha 2 chain *(COL1A2)*, bone sialoprotein 2 *(IBSP)* gene expression, accompanied by elevated numbers of bone nodule development and heightened level of osteocalcin *(OCN)* protein expression [31]. Our findings concur with the foregoing as we noted a significant upregulation of miR-129-5p expression in Jagged1-treated PDLSCs, similar to that of BMMSCs. However, in other cell types, such as in adipose-derived stem cells (ADSCs), the overexpression of miR-129-5p led to different outcomes. For instance, in miR-129-5p stimulation of ADSCs significantly reduced mineral nodule formation, decreased the ALP activity and suppressed the relative expression of *RUNX2*, osterix *(OSX)* and *OCN* [32].

The upregulation of miR-192-5p expression is known to correlate well with cell proliferation in diverse cell types, particularly tumorigenic cells. In hepatocellular carcinoma, for instance, miR-192-5p promoted the proliferation and metastasis of the tumor cells by targeting semaphorin 3A (SEMA3A) [33]. Furthermore, the overexpression of miR-192-5p could suppress the M1 macrophage polarization by targeting epiregulin (EREG) in gouty arthritis mice, leading to reduced ankle joint swelling, decreased synovial inflammatory cell infiltration and ameliorating bone erosion [34]. Therefore, in translational terms, promotion of cellular proliferation and reduction of inflammation through miRNA activity could offer remarkable clinical benefits, particularly periodontal tissue regeneration, where cell insufficiency and inflammation are the major drawbacks.

It is noteworthy that both upregulation as well as downregulation of several miRNAs and differential patterns of osteogenesis have been reported in different Jagged1-exposed cell types by a number of researchers [35–37]. Thus, Li et al. [35] found overexpression of mhesiR-133a-3p markedly inhibited the expression of osteogenesis-associated genes such as *RUNX2* and *OSX*, reduced ALP activity and mineralization by targeting ankyrin repeat domain 44 (ANKRD44) in BMMSCs. Similarly, Deng et al. [36] observed significant inhibition of osteogenic differentiation, cell migration, and cell proliferation in MC3T3-E1 cells due to miR-210-3p. This suppression was attributed to the direct targeting of brain-derived neurotrophic factor (BDNF) by miR-210-3p. On the contrary, Mollazadeh et al. [37]. found that the overexpression of miR-148b-3p could increase the osteogenic differentiation of human BMMSCs by enhancement of mineralized nodule development and increased osteoblastic cell differentiation.

Our results suggest that miRNAs may have dual functions in osteogenesis, either enhancing or inhibiting it, depending on the cell lineage. This underscores the critical role of cell type in determining miRNA function. As previously mentioned, the contrasting results in osteogenesis appear to be induced by the identical miRNA across different stem cell lineages, including BMMSCs, ADSCs, and finally, in PDLSCs, as shown in the present study [30–32]. These studies also revealed modulation of the Wnt signaling pathway, which produced contradictory outcomes. This duality in pathway function, capable of either inducing or inhibiting osteogenesis, appears to be directed by different mechanisms depending on the cell types involved [38, 39].

Previous research has indicated the promotion of osteogenic differentiation in hPDLs through the regulation of Notch receptor and target gene expression via the TGF-β signaling pathway [40]. We noted this phenomenon in our study as altered miRNAs from Jagged1-treated PDLSCs appeared to target the TGF-β signaling pathway. Additionally, miR-192-5p which significantly upregulated in Jagged1-treated cells appeared to target the Wnt signaling pathway. TGF-β, a pleiotropic cytokine abundantly seen in bone remodeling ecosystems has also been extensively studied for its contribution to proliferation and differentiation of human PDLSCs [40]. The Wnt signaling pathway a signal transduction pathway is also known to be crucial for osteogenic differentiation and bone formation in MSCs, playing a role in periodontal homeostasis, pathological process, and tissue regeneration [41]. In hPDLSCs, Wnt signaling pathway served as a key regulator of osteogenic differentiation [42]. Our results reiterate the importance of miRNA regulation of these pathways in controlling osteogenesis associated with periodontal regeneration.

In addition, our study revealed that Jagged1 upregulated miR-129-5p expression, targeting the Hippo signaling pathway in PDLSCs (*p* < 0.05). Hippo- Yes-associated protein (YAP) signaling pathway plays a crucial role in various biological processes, including tissue regeneration, regulation of cellular properties, such as proliferation and fate, by inducing gene expression through transcriptional enhancer associated domain transcription factors (TEADs) [43]. For instance, An et al. [44] noted that stimulation of the Hippo signaling pathway in BMSCs suppressed osteogenic differentiation. Additionally, *SOX2*, a *YAP1* target, had a role in maintaining stemness and constraining osteogenic differentiation through Dkk1-mediated suppression in the Wnt signaling pathway [45]. In cancer studies, miR-129-5p directly suppressed *YAP* and *TAZ* expression, leading to TEAD inactivation and Hippo signaling downregulation [46]. However, further research is warranted to assess the direct targets of miR-129-5p and whether its overexpression leads to Hippo signaling downregulation through YAP-TAZ, potentially promoting osteogenesis.

Nevertheless, it is important to acknowledge several limitations in our study. Firstly, this research is confined to an in vitro setting, which may not fully replicate the complexity of in vivo periodontal tissues. Primary cell culture under laboratory-controlled conditions might introduce artificial stimuli or biases that could influence miRNA patterns. Additionally, interactions with neighboring cell types, extracellular matrix components, and systemic factors are crucial but absent in in vitro studies, potentially limiting the clinical relevance of our findings. Lastly, our study employs static cell culture conditions for a specific duration. However, Notch signaling is a dynamic process that may exhibit fluctuations in activity over time, potentially hindering our understanding of how Notch activation influences miRNA expression patterns.

In conclusion, our findings revealed several markedly altered miRNAs in Jagged1-treated PDLSCs, linked to essential cellular pathways, particularly those regulating stem cell pluripotency. These miRNAs are predicted to target genes primarily involved in nucleotide binding, influencing diverse cellular processes and molecular functions. Further elucidation of novel biological roles of these miRNAs through Jagged1-Notch signaling holds promise in revolutionizing the landscape of regenerative medicine and dentistry.

## Data Availability

The datasets generated and/or analyzed during the current study are available from the corresponding author on reasonable request.
